# Prevalence and Determinants of the Double Burden of Malnutrition in Iraq: A Cross-Sectional Study

**DOI:** 10.7759/cureus.82565

**Published:** 2025-04-19

**Authors:** Dlkhwaz A Hama, Zana B Najmadden, Kaihan H Hama Salih, Huda J Mhamad

**Affiliations:** 1 Food Science and Quality Control, Halabja Technical College, Sulaimani Polytechnic University, Halabja, IRQ; 2 Research Center, University of Halabja, Halabja, IRQ

**Keywords:** child health, double burden malnutrition, iraq, malnutrition, nutritional status, obesity, overweight, wasting

## Abstract

Malnutrition in children mirrors socioeconomic status and plays a key role in health, economic, and social impacts. The double burden of malnutrition has attracted considerable attention from healthcare institutions and organizations. For the past three decades, studies on malnutrition have been restricted to focusing on only undernutrition or overnutrition. This study aimed to determine the dual burden of malnutrition and its contributing factors in Iraq. A cross-sectional study was conducted to determine the prevalence and contributing factors of malnutrition in 646 kindergarten children across the country. A questionnaire was used to collect data on the participants and their families. Data were analyzed using IBM SPSS Statistics for Windows, Version 22 (Released 2013; IBM Corp., Armonk, New York). The chi-square test was used to assess the relationship between variables and both forms of malnutrition, and statistical significance was evaluated at p < 0.05.

Results showed that wasting was found in 18 children (2.8%). Five-year-old children were more likely to be wasted than four-year-olds, and those who began complementary feeding after six months had higher rates of wasting. Children who received mixed feeding were more wasted compared to those who were breastfed or bottle-fed. Additionally, children of housewife mothers and those from middle-income families were more likely to experience wasting than those from low- or high-income families.

In addition, 48 children (7.5%) were found to be overweight. There was no statistically significant difference between the sexes. Five-year-olds were more affected than four-year-olds, and those who received mixed feeding were more affected than those who were exclusively breastfed or bottle-fed. Children of mothers who had completed primary or secondary education were more likely to be overweight, although no statistically significant difference was found based on parental education level or maternal employment status. Furthermore, children who began complementary feeding after six months and those from middle-income families were more likely to be overweight.

These findings contribute to a better understanding of the prevalence and contributing factors of both forms of malnutrition.

## Introduction

Children are exposed to different risks that greatly impact their current and future health status. Non-communicable diseases greatly contribute to these hazards, and nutritional imbalances are the main inducers, i.e., nutrition-related. Malnutrition is an important measure of development and shows the community's socioeconomic status. As it is widespread, malnutrition has huge morbidity and mortality. It greatly affects health, economic, and social impacts, and these effects are lasting and continuous. Globally, each year, 5 million children die owing to the impact of malnutrition [1]. Furthermore, various adult diseases are closely associated with malnutrition and improper nutrition in infancy. At the same time, proper nutrition in childhood is associated with favorable physical, mental, social, and even economic outcomes [2]. Malnutrition has various forms, including undernutrition, micronutrient deficiencies, and overnutrition. Undernutrition is the main point in diet-related agendas, specifically in low- and middle-income countries. However, urbanization and economic development lead to a nutritional transition to energy-dense foods. Regardless of this change, there are concurrent economic shortages, and nutritional deficiencies remain challenging for children. So, there is a co-occurrence of over- and undernutrition, shifting the burden to the dual burden of malnutrition (DBM), and it becomes a global health issue due to high social, economic, and health consequences [3].

According to the World Health Organization (WHO), DBM is "defined by the coexistence of undernutrition along with overweight, obesity, or diet-related non-communicable diseases (NCDs), within individuals, households, and populations" [4]. DBM can be seen at three different levels: individual level, which means the coexistence of undernutrition (wasting, stunting, or micronutrient deficiency) with overweight or obesity at a specific time or any point along his or her life; or the existence of under- or overnutrition within two different members of the same household; or at the community level, where both forms of malnutrition are common within the same population unit [5].

Undernutrition in all forms (wasting, stunting, micronutrient deficiencies) continuously poses individuals, families, communities, and countries with remarkable health problems. Although everyone is vulnerable to suffering from DBM, preschool children are a more susceptible population group [6]. In developing countries, one in five children suffers from undernutrition, with nearly half of deaths related to undernutrition [7]. Recent data show that the global prevalence of undernutrition is 6.8% [8], and 190 million children live with thinness [9].

At the same time, the rate of overweight and obese children is evolving unexpectedly. According to WHO, globally, in 2022, 390 million children were overweight, including 160 million who were obese. Among these, 37 million were children below five, and an additional 93 million were at risk of being overweight [9]. In the same year, the prevalence of obesity in children under five was 5.6% [8]. The prevalence of obesity has been increasing worldwide, especially in the last two to three decades, with developing countries showing a relatively greater increase than developed countries [10]. The Eastern Mediterranean Region is among the areas with the highest ratio of overweight children. In Iraq, the prevalence of overweight was 20% [11].

Importantly, overweight and obesity affect more young children, or even begin before childhood [12]. This increases the risk of obesity in adulthood and its associated health effects. These individuals tend to experience more complicated forms of obesity than those who become obese later in life. Consumption of high-energy foods, lifestyle changes, urbanization, and sedentary behaviors such as watching games and TV are the leading causes of obesity in this region [11]. In response, in 2016, WHO developed double-duty actions to establish a framework that includes policies, interventions, and programs to reduce the burden of DBM.

A common method of evaluating the dual burden is to assess the prevalence of both overnutrition and undernutrition. The best indicators are anthropometric measurements [3].

Anthropometric measures are practical ways of assessing growth in children. Indicators from these measurements can be used to identify children at risk of under- or overnutrition. For instance, anthropometric values such as weight-for-height z-scores in children are commonly used to define DBM [4].

Iraq is one of the countries in the Eastern Mediterranean Region. Iraqi society has endured many challenges over the last three decades, including various economic and political issues, making it valuable to understand the drivers of malnutrition in children.

This study aims to identify the prevalence of DBM in children attending kindergartens in the Halabja governorate of Iraq, as well as to identify predictive factors of DBM in these susceptible groups in the population.

## Materials and methods

Study design and setting

This study is a descriptive, cross-sectional study. The data were collected from 646 children aged 4 and 5 years attending 10 kindergartens in Halabja city during the period from March 1 to May 1, 2024. This study was conducted in Halabja governorate, which is situated in the northeast of Iraq. Halabja is a newly established, small governorate that separated from Sulaimaniyah Governorate in 2014. The governorate consists of the capital city, Halabja, and the districts of Biyare, Xurmal, Bemo, and Sirwan, with an estimated population of 140,000. The inhabitants of the governorate are Kurdish, speaking the Sorani and Hawrami dialects [13].

Tools and data collection

This study assessed the children's nutritional status and health characteristics using a structured questionnaire. The questionnaire was designed by a group of experts, including pediatricians and dietitians. Anthropometric measurements were used to assess the nutritional status of all participants. Weight and height were measured according to standard guidelines. The most common indicators of DBM at the community level were used, defined as the coexistence of overweight/obesity (OW/OB) and thinness/wasting/undernutrition (TWU) [4]. Before conducting the study, the purpose was explained to the General Directorate of Education of the governorate, and permission was obtained. The researchers then visited the kindergartens and explained the purpose of the study to the staff and to the parents or health caretakers of the children. The children were divided into two age groups: four years and five years, with equal representation of both sexes within each age group. Ten kindergartens were randomly selected to represent the entire city of Halabja. The structured questionnaire was designed to gather information on various aspects of the children’s biographic, medical, and family backgrounds. Biographic data about the child included age and sex, birth weight (kg), type of feeding (breastfeeding, formula, etc.), age at the start of complementary feeding (months), and presence of chronic diseases or allergies. Biographic information about the family included the mother’s age, parents' education levels, parents' occupations, family size, and family income. The questionnaires were completed by the children’s parents after obtaining their informed consent. The purpose of the study was explained to them, and the researchers assisted in clarifying any points in the questionnaire. The collected data were entered into Microsoft Excel sheets (Microsoft Corporation, Redmond, Washington), and interpretation was performed as required.

Inclusion criteria

Children from public kindergartens of Halabja governorate, aged four to five years, who are healthy and have no chronic diseases such as hypothyroidism, short stature, or congenital heart diseases, and have no dysmorphic features; Kurdish ethnicity.

Exclusion criteria

Any child with a chronic disease, attending private kindergartens, living outside the governorate, whose parents refused to participate in the study, or of non-Kurdish ethnicity, was excluded.

Anthropometric measurements

Anthropometric measurements included weight, measured in kilograms using an electronic body scale placed on a flat and hard surface. Weight was recorded to the nearest 100 grams (0.1 kg). Height was measured while the child stood upright, with the headboard placed on the highest point of the head with enough pressure to compress the hair.

Definitions applied

Wasting indicates that a child is underweight for their height. It is one of the indicators of acute malnutrition in children and is measured as a z-score for weight-for-height less than −2 SD of the WHO Child Growth Standards median [14].

Overweight, for similar height and gender, usually reflects excess weight from muscle, bone, fat, water, or a combination of these, while obesity typically reflects excess body fat. Overweight is measured as a z-score for weight-for-height greater than +2 to less than or equal to +3 SD of the WHO Child Growth Standards median [15].

Family income was categorized into three levels for analysis: low income was defined as less than 400,000 IQD per month; medium income as between 400,000 and 1,000,000 IQD per month; and high income as more than 1,000,000 IQD per month.

Statistical analysis

Data were analyzed using IBM SPSS Statistics for Windows, Version 22 (Released 2013; IBM Corp., Armonk, New York). The chi-square test was used to determine the relationship between variables and both forms of malnutrition. Statistical significance was evaluated at a p-value of less than 0.05.

Ethical approval and informed consent

Before conducting the study, the ethical committee at Halabja University approved the research protocol (Reference number 03 2024/6). Written informed consent was obtained from the parents of the children after explaining the purpose of the study.

## Results

This study provides a foundation for understanding the nutritional status of children in kindergartens in the Halabja governorate. The study population included 646 students from 10 kindergartens in Halabja city. Among them, 310 (48%) were male and 336 (52%) were female. A total of 78 children (12.1%) were four years old, while 568 (87.9%) were five years old. Tables 1, 2 present the characteristics of the screened children, including details from infancy and information about their parents.

**Table 1:** Distribution of children in kindergartens of Halabja Governorate according to their gender, age groups, type of feeding, complementary feeding, and gestational age. The WHO classifies newborns into term and preterm categories. *Preterm babies are further classified based on gestational age as follows: extremely preterm (<28 weeks), very preterm (28–32 weeks), and moderate to late preterm (32–37 weeks). For ease of interpretation, we grouped them into two categories: <28 weeks and 28–37 weeks.

Variable	Frequency (n)	Percentage (%)
Gender		
Male	310.0	48.0
Female	336.0	52.0
Age groups		
4 years	78	12.1
5 years	568	87.9
Type of feeding		
Breastfeeding	229	35.4
Bottle feeding	93	14.4
Mixed feeding	324	50.2
Gestational age*		
9 months	556	86.0
Before 9 months	84	13.0
7 months	6	0.93
Complementary feeding		
Before 6 months	129	20.0
After 6 months	517	80.0

**Table 2:** Biographic information of parents of children in kindergartens in Halabja Governorate. *Low income = <400,000 IQD per month; Medium income = between 400,000 and 1,000,000 IQD per month; High income = >1,000,000 IQD per month.

Variables	Frequency (n)	Percentage (%)
Mothers age group		
15-24	20	3.1
24-35	360	55.7
35-45	234	36.2
≥45	32	5.0
Mothers’ education		
Illiterate	6	6.0
Primary and secondary school	281	43.5
Institute	167	25.9
College	152	23.5
High education	7	1.1
Fathers’ education		
Illiterate	12	1.9
Primary and secondary school	341	52.8
Institute	128	19.8
College	149	23.1
High education	16	2.5
Mothers’ occupation		
Housewife	436	67.5
Employee	200	31.0
Other	10	1.5
Family size		
3-5 persons	516	79.9
6-9 persons	129	20.0
≥9 persons	1	0.1
Family socioeconomic status*		
Low	111	17.2
Medium	373	57.7
High	162	25.1

Wasting

Wasting and Characteristics of the Participants

Looking at Table 3, it is apparent that the prevalence of wasting, determined by a Z score less than −2 according to WHO guidelines, was 2.8%. All results related to wasting and the characteristics of the children are shown in Table 3. According to these results, 18 children had wasting: 10 were male (1.3%) and 8 were female (1.2%), with no significant difference between the sexes. Regarding age, 17 were five-year-old children, indicating a higher prevalence compared to four-year-olds. With respect to feeding, although there was no statistically significant difference between types of feeding, children with mixed feeding were more affected than those who were breastfed or bottle-fed. Importantly, a positive correlation was found between wasting and initiation of complementary feeding after six months (2.2%).

**Table 3:** Association between nutritional status (assessed by WHZ) and children's gender, age groups, type of feeding, complementary feeding, and gestational age in kindergartens of Halabja Governorate. * WHZ (weight-for-length/height z-score) is a WHO measure to assess the nutritional status of children; it has a specific value for each sex.

	WHZ*									
	Wasting		Normal		Overweight		Total		Chi-square	P value
	N	%	N	%	N	%	N	%		
Gender										
Male	10	1.5	279	43.2	21	3.3	310	48.0	2.688	0.616
Female	8	1.3	301	46.6	27	4.2	336	52.0		
Total	18	2.8	580	89.8	48	7.5	646	100		
Child age group										
4 years	1	0.2	73	11.3	4	0.7	78	12.1	2.620	0.787
5 years	17	2.7	507	78.5	44	6.8	568	87.9		
Total	18	2.8	580	89.8	48	7.5	646	100		
Type feeding										
Breast	7	1.1	209	32.4	13	2	229	35.4	14.355	0.116
Bottle	2	0.3	77	11.9	14	2.2	93	14.4		
Mixed	9	1.4	294	45.5	21	3.2	324	50.2		
Total	18	2.8	580	89.8	48	7.5	646	100		
Complementary food										
Before 6 months	4	0.6	117	18.1	8	1.3	129	20	0.400	0.803
After 6 months	14	2.2	463	71.7	40	6.2	517	80		
Total	18	2.8	580	89.8	48	7.5	646	100		
Birth date										
9 months	13	2	504	78	39	6	556	86.1	5.237	0.163
Before 9 months	5	0.8	71	11.0	8	1.2	84	13.0		
7 months	0	0.0	5	0.8	1	0.2	6	0.9		
Total	18	2.8	580	89.8	48	7.5	646	100		

Wasting and Demographic Data of the Parents

Concerning wasting with the parents' age, level of education, occupation, and family socioeconomic status, the results are shown in Table 4. Our results showed that children of mothers with primary and secondary level education are more wasted (1.2%) than children with other levels of education. Similarly, this is true for their fathers. Children of fathers who have primary and secondary school education are more wasted (1.5%) than those who have other levels of education, like institute, college, and higher education certificates. Mothers' age and occupation are important determinants of malnutrition. The current study showed that children of 25-45-year-old mothers are more wasted (1.4%) than children of younger or older mothers. In addition, children of housewife mothers are more wasted (2.2%) than children with employed mothers. Concerning the family's socioeconomic status, the rate of wasting children in middle-, low-, and high-income families is 1.5%, 0.9%, and 0.3%, respectively. Furthermore, there was no statistically significant difference between full-term and preterm babies.

**Table 4:** Association between nutritional status (assessed by WHZ) and parents' level of education, mother's occupation, mother's age, family size, and family socioeconomic status among children in kindergartens of Halabja Governorate. WHZ: weight-for-length/height z-score.

Variables	WHZ											
	Wasting			Normal		Overweight		Total			Chi-square	P value
	N		%	N	%	N	%	N		%		
Mother education												
Illiterate	2		0.3	31	4.8	6	0.9	39		6.0	6.545	0.421
Primary and secondary school	8		1.2	256	39.6	17	2.6	281		43.5		
Institute	4		0.6	152	23.5	11	1.7	167		25.9		
College	4		0.6	135	20.9	13	2.0	152		23.5		
High education	0		0.0	6	0.9	1	0.2	7		1.1		
Total	18		2.8	580	89.8	48	7.5	646		100		
Fathers’ education												
Illiterate	0		0.0	12	1.9	0	0.0	12		1.9	4.739	0.942
Primary and secondary school	10		1.5	303	46.9	28	4.3	341		52.8		
Institute	5		0.8	115	17.8	8	1.2	128		19.8		
College	3		0.5	134	20.7	12	1.9	149		23.1		
High education	0		0.0	16	2.5	0	0.0	16		2.5		
Total	18		2.8	580	89.8	48	7.5	646		100		
Mothers’ occupation												
Housewife	14		2.2	394	61.0	28	4.3	436		67.5	6.508	0.153
Employee	3		0.5	177	27.4	20	3.1	200		31.0		
Other	1		0.2	9	1.4	0	0.0	10		1.5		
Total	18		2.8	580	89.8	48	7.5	646		100		
Mothers’ age group												
15-24		0	0.0	18	2.8	2	0.3	20	3.1		2.207	0.880
24-35	8		1.2	325	50.3	27	4.2	360		55.7		
35-45	9		1.4	208	32.2	17	2.6	234		36.2		
≥45	1		0.2	29	4.5	2	0.3	32		5.0		
Total	18		2.8	580	89.8	48	7.5	646		100		
Family size												
3-5 persons	11		1.7	469	72.6	36	5.6	516		79.9	5.248	0.181
6-9 persons	7		1.1	110	17.0	12	1.9	129		20.0		
≥9 persons	0		0.0	1	0.2	0	0.0	1		0.2		
Total	18		2.8	580	89.8	48	7.5	646		100		
Family socioeconomic status												
Low	6		0.9	100	15.5	5	0.8	111		17.2	10.590	0.037
Medium	10		1.5	327	50.6	36	5.5	373		57.7		
High	2		0.3	153	23.7	7	1.1	162		25.1		
Total	18		2.8	580	89.8	48	7.5	646		100		

Overweight

Overweight and Characteristics of the Participants

According to the results shown in Table 3, the prevalence of overweight children was 7.5%. Females (4.2%) were affected more than males (3.3%), although there was no statistically significant result. About 6.8% of them were in the five-year-old group, while only 0.7% were four years old. Regarding the type of feeding during infancy, nearly 50% of overweight children had mixed feeding, followed by bottle and breast feeding. An important issue that emerged from the data was that the majority of overweight children started complementary feeding after six months, while a minority of them started before six months (2.2% and 0.6%, respectively).

Overweight and Demographics of the Parents

Concerning overweight children with parental characteristics, children of mothers with primary and secondary levels of education were more overweight (2.6%) than children of mothers with college and institute levels of education. Similarly, children of fathers with primary and secondary levels of education were more overweight than those of fathers with college and institute levels of education. The results of this study show that there was no statistically significant difference between housewife mothers and employed mothers who had overweight children. Furthermore, the age of the mothers of most overweight children was between 24-35 years (1.4%), followed by mothers aged 35-45 years (1.2%). Family income has a substantial role in a child’s nutritional status. According to the results shown in Table 4, families with middle income had more overweight children than those from high-income families.

## Discussion

This study set out with the aim of assessing the prevalence and the determinant factors of the DBM in a newly established governorate in Iraq. Our results show that the prevalence of wasting is 2.8%, which is lower than the global rate of wasting (6.8%), as reported by WHO, UNICEF, and FAO in 2024 [8]. Similarly, it is lower than 12.6% in Southeast Asia [14] and 7.5% in the EMR [16]. Regarding neighboring countries, it is lower than the prevalence of wasting in Iran [17] and Saudi Arabia [18], and it is also lower when compared with previous studies in Iraq [16]. One anticipated finding was that five-year-old children were more affected than four-year-olds, in contrast to the study by Fuad et al. in Egypt in 2023, which found that children younger than five years were the most affected group [15]. This study shows that the highest prevalence of wasting was found in breastfed and mixed-fed children, in contrast to the study by Al Shameri et al. in 2022 [16]. This may be explained by the possibility that mothers had comorbidities or micronutrient deficiencies that adversely affected the growth of their children. One interesting finding is that the majority of wasting was among children who started complementary feeding after six months of age. Delayed introduction of essential nutrients during this critical period may adversely affect growth in subsequent stages. Family size is an important determinant of undernutrition, as children in crowded families are more susceptible to malnutrition due to reduced healthcare access and limited nutritional intake, as shown in previous studies [19]. However, we did not find a significant relationship between undernutrition and family size. Parental literacy is among the important determinants of nutritional status, as socioeconomic status directly relates to health outcomes such as wasting. This has been reported in previous studies [16]. Although we could not find such a relationship between wasting and parental education level in our study, the reasons remain unknown.

In this study, the prevalence of overweight was 7.4%, higher than the global prevalence of 6.1% in children under five, according to the most recent WHO report in 2024 [20], but lower when compared with the prevalence of overweight in New Zealand, Australia [8], Egypt [15], and Saudi Arabia [21]. This study reports a higher prevalence of overweight compared to updated reports in Iraq [8]. This may be attributed to the nutritional transition toward energy-dense foods, sedentary lifestyles such as watching television and playing electronic games, and urbanization. This is consistent with the global increase in the prevalence of overweight in children [11]. The current study found that bottle-fed and mixed-fed infants were more likely to be overweight than breastfed infants. This is consistent with the findings of Arif [11], as breast milk contains essential nutrients that help prevent overweight and obesity. Regarding family income, this study shows no significant differences among income groups, suggesting that all children are at risk in the absence of appropriate interventions. This finding is similar to that of Arif [11]. A study from the United States also shows that disadvantaged children are more susceptible to overweight and obesity than advantaged children [22]. On the topic of complementary feeding and overweight, this study shows that delayed introduction of complementary feeding is associated with overweight. This is similar to another study from the United States, which found that exclusive milk feeding during the first six months is associated with an increased risk of being overweight later on [23]. This may be due to a common misconception among families that after six months of age, the child can consume everything, which may lead to overfeeding and subsequent overweight.

This study did not find a significant relationship between parents’ education and childhood overweight, similar to the findings of Li et al. in 2024 [24], but in contrast to the studies of Seum et al. in Germany [25] and Hsu et al. in Taiwan [26], which found an inverse relationship between parental education and childhood overweight and obesity. This discrepancy may be due to urbanization and the rapid demographic and epidemiological changes occurring in the region and the country, leading to shifts in diet and body composition across all children. Regarding maternal occupation, this study found no significant relationship between employed and unemployed mothers, in contrast to the findings of Li et al. [24], who reported that children of employed mothers were more likely to be overweight than those of non-employed mothers. This may be explained by the fact that working hours in our country are shorter than in other regions due to the large number of official holidays and changing work schedules caused by instability in climate conditions. The current study found that high- and middle-income families had more overweight children than low-income families, which is consistent with findings from other studies conducted in Taiwan [26]. However, recent studies have pointed out that all children, regardless of family income, may be at risk of being overweight [11, 27].

Finally, several important limitations should be considered. First, the questionnaire used for data collection lacked formal testing for validity and reliability. Second, the data were collected from 10 kindergartens, while there are 20 public and private kindergartens in the governorate. Lastly, responses to some questions, such as those related to family income, may be biased, as they involve personal information.

## Conclusions

The purpose of the current study was to determine the dual burden of malnutrition in Iraq, its prevalence, and determinant factors. This study has shown that a significant proportion of Iraqi children suffer from both forms of malnutrition, although being overweight is more prevalent than wasting. The prevalence of wasting was 2.8%, and it was more common in children who started complementary feeding after six months of age. Additionally, 7.5% of the children were overweight, and it was more common in those who had mixed feeding and started complementary feeding after six months of age. Importantly, the added burden of overweight exaggerates the problem of malnutrition in our communities with limited resources. These findings provide insights for healthcare providers, and healthcare policies should prioritize early complementary feeding education and promote exclusive breastfeeding. These findings enhance our understanding of the contributing factors of both forms of malnutrition. More research is required to better understand the determinant factors of malnutrition in children. In addition, these findings have several practical implications.

## Figures and Tables

**Figure 1:**
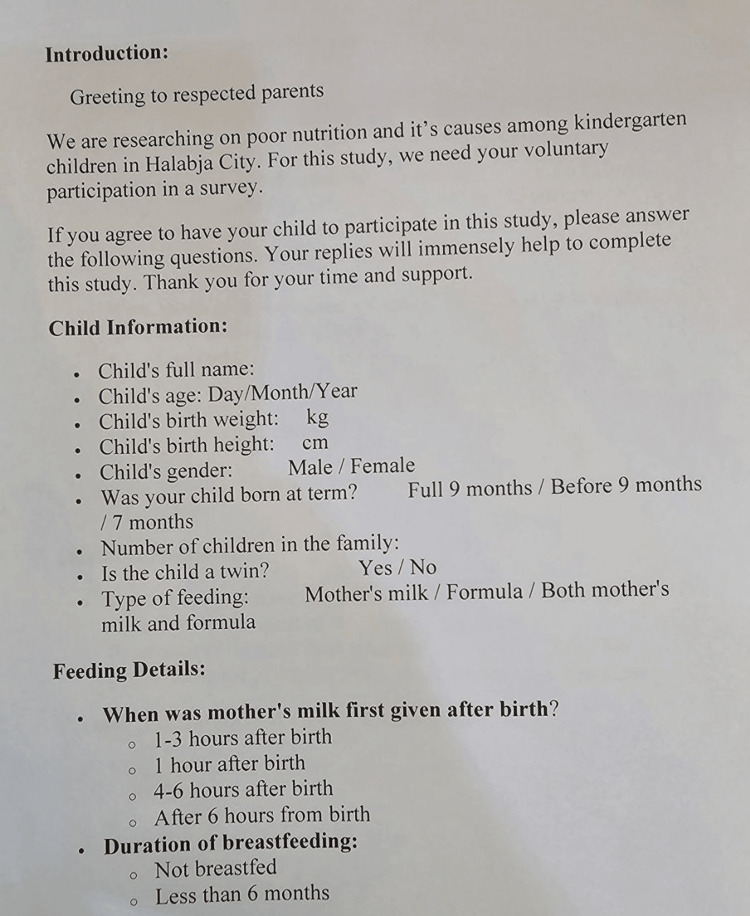
The questionnaire used in this study (Part A)

**Figure 2:**
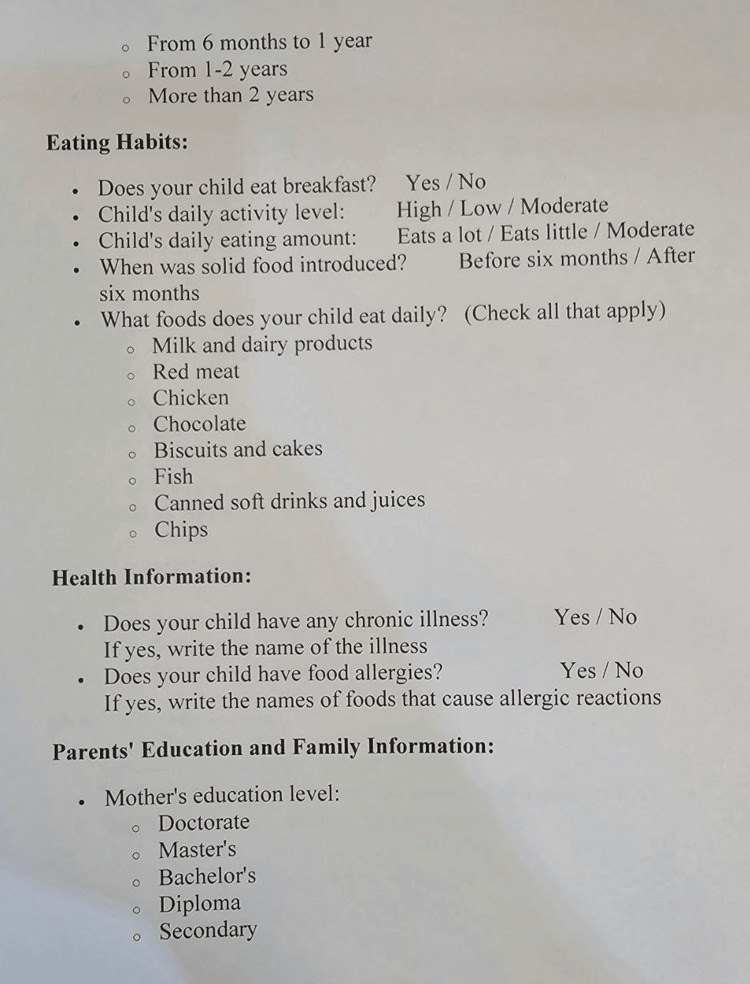
The questionnaire used in this study (Part B)

**Figure 3:**
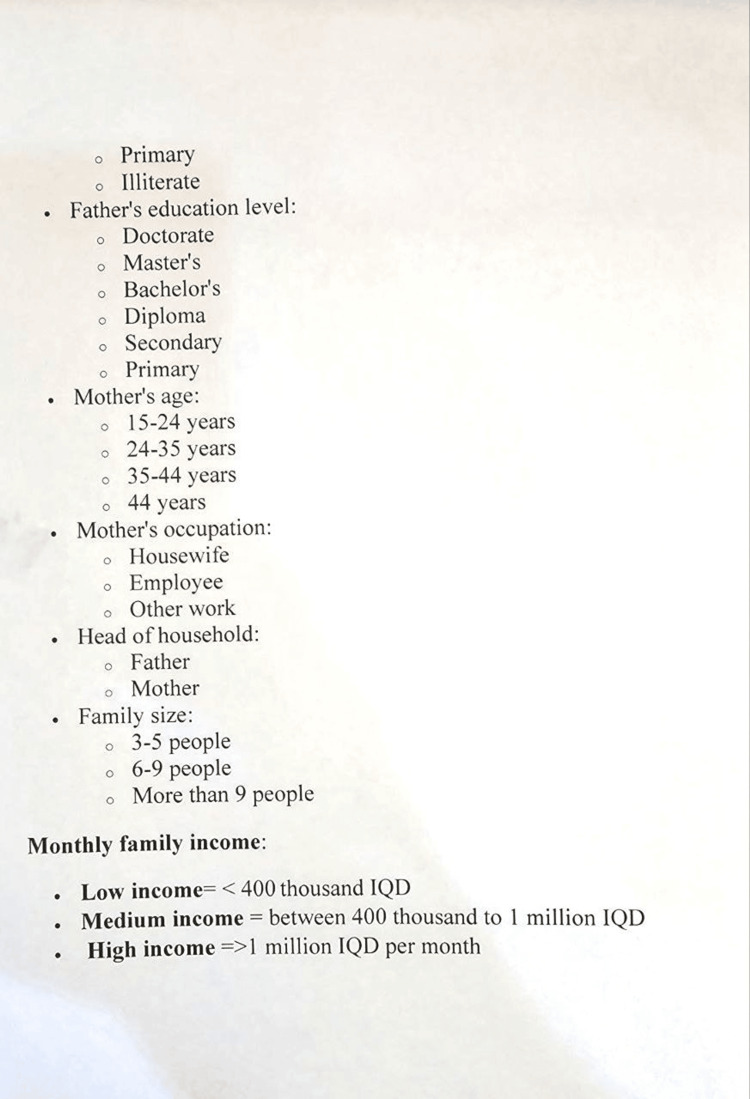
The questionnaire used in this study (Part C)
